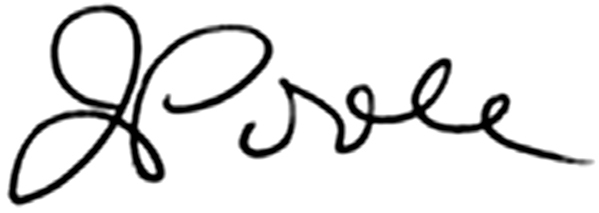# State of the Journal 2024: *Heart Rhythm O*^*2*^

**DOI:** 10.1016/j.hroo.2023.12.005

**Published:** 2024-01-22

**Authors:** Jeanne E. Poole

Greetings from *Heart Rhythm O*^*2*^ (*HRO2*). The Journal is starting its 5th year of publication, and we are looking forward to continued growth. As you know, we have been publishing monthly now for the past year. Submissions continue to be strong, with an acceptance rate of 52%. The majority of articles published are original research articles. The 2 most frequently downloaded original articles were a Design Paper, “A randomized controlled trial of pulsed field ablation versus standard-of-care ablation for paroxysmal atrial fibrillation: the ADVENT trial rationale and design,” by Vivek Y. Reddy et al,^1^ and a Research Letter, “Low-level tragus stimulation improves autoantibody-induced hyperadrenergic postural tachycardia syndrome in rabbits,” by Ankur N. Shah et al.^2^ We also published 8 review articles covering broad topics. The most frequently downloaded review article was “Cardioneuroablation: where are we at?” by Jose Carlos Pachon and colleagues.^3^

A popular article type is our *Perspectives in Contrast,* which features 2 opposing views on therapeutic or diagnostic topics. In the February issue, Dr Jan De Pooter^4^ and Drs Shunmuga Sundaram Ponnusamy and Pugazhendhi Vijayaraman^5^ defended their favored type of lead for physiological pacing—either stylet-driven^4^ or lumenless.^5^

This past year, we welcomed 2 new social media editors: Valentina Kutyifa and Nashwa Abdulsalam. With our busy lives, social media conversations are a great way to quickly assimilate new science and engage with peers in a post-publication analysis. Currently, *HRO2* has more than 3600 followers. Please join in and help us to highlight your favorite articles.

We believe it is important that *HRO2,* although based in the United States, is also a home for international researchers and readers. International articles represent 51% of submissions, with the majority in 2023 from Japan, The Netherlands, and China. Downloads also are globally representative, with 19% from India and 26% from Europe. Extending our emphasis on global participation, we are highlighting in our *Global Voices* section the early seminal contributions to electrophysiology from our Latin American and Israeli colleagues. We will continue this series through 2024.

*HRO2* received its first impact factor this year, a respectable 1.9. As you all know, the Clarivate Impact Factor calculation is based on the average number of times articles published in the journal over the previous two years were cited in the current year. The Clarivate Impact factor is just one measure of a journal’s success. Additional analytics include H-Index, 9; Elsevier CiteScore, 1.8; and Scimago Journal & Country Rank, 0.71 (ranking 130 of 367 journals in cardiology and cardiovascular medicine). Examination of the top cited articles across journals is also helpful to gauge what topics are of greatest interest as well as to stay abreast of the latest innovations and discoveries. In the future, traditional analytics may compete with social media “hits” as what best reflects the popularity and importance of any individual journal. With your support, *HRO2* will continue to grow, bringing relevant science to our global readership.

*HRO2* is a Gold Open Access journal, joining many journals that have been launched as Gold in the past few years. You may have questions about where “open access” is headed. The foundational concept is based on the accepted belief that science should be freely and immediately available to everyone. In the traditional model, publishing houses assume the full cost of the publication process, and publications were immediately available only to those with a subscription or after a lengthy embargo period. Green Open Access satisfies one part of the transitional concept—that accepted papers are made available, hosted on a freely accessible open access platform. The National Institutes of Health (NIH) has notified grant applicants that, by 2026, all manuscripts funded by the NIH must be hosted on an open access server. Gold Open Access extends the transformational change by exchanging the subscription model to the authors to cover the article publishing charges. As academic institutions and libraries increasingly abandon journal subscriptions, it is anticipated that the cost of publishing will be incorporated into grant funding, designated faculty budgets, or other sources available to the authors.

Finally, on behalf of myself and our stellar Heart Rhythm Society Journal staff, we would like to thank our editors, reviewers, and authors for helping to make our first 4 years successful. We wish you all a Happy and Healthy New Year!

Jeanne E. Poole, MD, FHRS

Editor-in-Chief, *Heart Rhythm O*^2^

**Figure:**